# The maternal and child mortality in the Middle East and North Africa between 2000 and 2020: the role of health financing

**DOI:** 10.1186/s41256-025-00459-1

**Published:** 2025-10-30

**Authors:** Tianjiao Gao, Marwa Farag, Guohong Li, Wu Zeng

**Affiliations:** 1 Department of Global Health, Georgetown University, Washington, DC USA; 2 School of Public Health, University of Saskatchewan, Saskatoon, SK S7N 4Z2 Canada; 3 School of Public Administration and Development Economics (SPADE), Doha Institute for Graduate Studies, Doha, Qatar; 4 School of Global Health, Shanghai Jiaotong University School of Medicine, Shanghai, China; 5 Heller School for Social Policy & Management, Brandeis University, Waltham, MA USA

**Keywords:** Maternal health, Child health, Health financing, Middle East and North Africa

## Abstract

**Background:**

Improving maternal and child health (MCH) outcomes is a critical agenda item in global development. Health financing factors play a crucial role in affecting MCH outcomes, which vary substantially in the Middle East and North Africa (MENA) region. This study aims to examine the trends in maternal mortality rate (MMR), infant mortality rate (IMR), and under-5 mortality rate (U5MR) in the MENA region and the potential impact of health financing factors on them.

**Methods:**

We compiled data on MCH mortalities and potential determinants, including health financing factors, for all countries in the MENA region from 2000 to 2020. We calculated the growth rate of mortalities and explored the association between mortality rates and potential determinants using fixed-effects models.

**Results:**

The average MMR, IMR, and U5MR showed an overall descending trend in the region. Middle-income countries experienced the highest reduction rates (3.46–3.73%), followed by high-income countries (2.97–3.02%) and then low-income countries (0.33–0.92%). Gross domestic product (GDP) per capita, current health expenditure (CHE) per capita, urbanization, and fragility were consistently associated with all three mortality rates (*p* < 0.05). GDP elasticity of MMR, IMR, and U5MR was estimated at − 0.121, − 0.076, and − 0.138, respectively, while corresponding CHE elasticity was − 0.319, − 0.275, and − 0.225, with a larger magnitude. Fragility was positively associated with higher MMR, IMR, and U5MR. Additionally, government health spending, air pollution, and immigration were associated with MMR, but not with IMR and U5MR.

**Conclusions:**

Low-income countries in the MENA region, with the highest mortality rates, face greater challenges in reducing MCH mortality rates, necessitating tailored interventions to expand evidence-based MCH services and/or reinforce their effectiveness. Total investment in health plays a critical role in reducing mortality rates. Efforts to build a sustainable health financing system are key to improving MCH outcomes. Besides, endeavors to address broader socioeconomic factors and political stability should be prioritized in countries with major concerns of poverty and conflict.

**Supplementary Information:**

The online version contains supplementary material available at 10.1186/s41256-025-00459-1.

## Introduction

Maternal and child health (MCH) is a critical concern worldwide, including in the Middle East and North Africa (MENA) region. Significant advancements in improving MCH outcomes have been achieved in the region. In the 1960s, health conditions in MENA countries were characterized by low life expectancy and high child mortality rates. Over the past several decades, many countries in the region have made substantial efforts to improve health service delivery [1] and have made basic health services available to the public, substantially reducing infant and child mortality [2]. Consequently, the average infant mortality rate (IMR) plunged from 137 in 1970 to 21 per 1,000 live births in 2012 in the region [3]. Meanwhile, significant progress has been made in maternal health and life expectancy [4].

Despite ongoing efforts and progress in public health within the region, challenges remain in further reducing maternal and child mortality. First, some countries have witnessed the deterioration of MCH service delivery. In Yemen, more than half of the health facilities were destroyed between 2014 and 2021, and the rest could not meet the health needs of the entire population, limiting the quantity and quality of services provided for pregnant women [5]. Second, in some countries, maternal and child mortality rates have stabilized at low levels without significant improvement in recent years. For instance, Israel has maintained a maternal mortality rate (MMR) of 3 per 100,000 live births since 2008, with no subsequent decline [6]. Third, the limited capacity of service provision has prohibited the amelioration of MCH. Disparities in health outcomes and coverage of healthcare services persist between rural and urban populations across the region [1].

Health financing is an important factor in improving health outcomes, including reducing MCH-related mortality. Previous studies have suggested that a strong health system, particularly robust health financing, plays a crucial role in determining health outcomes. Strengthening health financing is proposed as a main approach to advancing a country towards universal health coverage (UHC) to reduce maternal and child mortality [7–11]. Total health expenditure is considered a major driver of the reduction in deaths. In a global study, Owusu examined the relationship between total health expenditure and maternal and infant mortality rates. The research showed that a 1% increase in current health expenditure (CHE) was associated with a 0.09–1.91% decrease in MMR and a 0.09–1.45% decrease in IMR [12]. Additionally, studies have also indicated that the structure of health financing affects MCH-related mortalities [13–15]. Kiross found that in Sub-Saharan African countries, public, private, and external health expenditures were all negatively associated with the neonatal mortality rate, though the magnitude of the impact varied [14]. Notably, government commitment to health, measured by domestic general government health expenditure as a share of general government expenditure, is essential for health improvement. An increase in government commitment to health has significantly increased the efficiency of health programs in low- and middle-income countries, thus improving health outcomes [16]. The impact of health financing on health outcomes, such as mortality, does not arise in isolation. Evidence shows that stronger health financing systems are associated with higher coverage and improved quality of essential MCH services (e.g., immunization and skilled birth attendance), which are critical for saving lives [17, 18].

The MENA region, with vastly diverse economic conditions and health systems, presents a unique platform for examining the role of health financing factors in improving MCH outcomes. This study leverages 21 years of data in the region to investigate MCH-related mortality trends across countries and examine the associated health financing drivers. We highlighted the role of health financing in reducing MCH-related mortality with empirical evidence. Beyond conventional determinants, we also extended the analytical framework by explicitly integrating the fragile and conflict-affected status of MENA countries, thereby capturing an often-overlooked dimension of health system vulnerability. The findings aim to inform the development of more effective health financing policies to enhance MCH outcomes in the MENA region.

## Methods

### Analytic framework

In this study, we started with a description of the trends in maternal and child mortality in the region and then used a regression model to examine the potential factors associated with the mortality rates. To select potential determinants of MCH outcomes, we followed a framework developed by the World Bank [19]. The framework categorizes potential determinants of MCH outcomes into four groups: (1) economic indicators, (2) health financing indicators, (3) demographic indicators, and (4) environmental indicators [20–24]. These four categories of determinants were a key subset of a broader framework that summarized the maternal health production process and encapsulated additional categories of culture and political system [25]. Given that this region includes countries experiencing prolonged conflicts, which might impede progress toward achieving their health goals, we also incorporated fragility/conflict-affected status as an additional factor influencing MCH outcomes.

### Measurements

The key measures of MCH outcomes are: (1) MMR, measured as the number of maternal deaths per 100,000 live births; (2) IMR, measured as the number of infant deaths per 1,000 live births; and (3) under-5 mortality rate (U5MR), measured as the number of deaths among children under five per 1,000 live births. We collected data on these indicators for each MENA country/territory from 2000 to 2020 from the World Development Indicators (WDI) database by the World Bank and the International Migrant Stock by the United Nations [26]. The names of the countries/territories are listed in Appendix A.

Following the World Bank framework to determine potential factors associated with MCH outcomes, we included a total of 10 indicators [27–29]. Socioeconomic status was measured by the gross domestic product (GDP) per capita. For health financing indicators, we included: (1) total health spending, measured by CHE per capita; (2) the structure of health spending, measured by domestic general government health expenditure as a share of current health expenditure (DGGHE/CHE), domestic private health expenditure as a share of current health expenditure (DPHE/CHE), and external health expenditure as a share of current health expenditure (EHE/CHE); and (3) government financial commitment to health, measured by domestic general government health expenditure as a share of general government expenditure (DGGHE/GGE) in the study. We used urbanization and immigration to measure the demographic indicators. Lastly, we included CO_2_ emissions to reflect the environmental status and fragility/conflict to measure political stability in the country. Table 1 provides detailed definitions of each independent variable.

**Table 1:** Definitions of independent variables

Category	Variable	Variable definition
Economy	GDP per capita	GDP per capita in current US$
Health financing	CHE per capita	Current health expenditure per capita in current US$
	DGGHE/CHE	Domestic general government health expenditure as a share of current health expenditure in percentage points (0–100)
	DPHE/CHE	Domestic private health expenditure as a share of current health expenditure in percentage points (0–100)
	EHE/CHE	External health expenditure as a share of current health expenditure in percentage points (0–100)
	DGGHE/GGE	Domestic general government health expenditure as a share of general government expenditure in percentage points (0–100)
Demography	Urbanization	Urban population as share of total population in percentage points (0–100)
	Immigration	The international migrant stock as a percentage of the total population (both sexes combined)
Environment	CO_2_ emissions	CO_2_ emission metric tons per capita
Fragile/conflict-affected status	Fragility	A country’s fragile situations (1 = fragile/conflict, 0 otherwise)

GDP: gross domestic product; CHE: current health expenditure; DGGHE: domestic general government health expenditure; DPHE: domestic private health expenditure; EHE: external health expenditure; GGE: general government expenditure

### Data sources

All data were collected for the period 2000–2020. The WDI database compiled by the World Bank served as the primary data source [6], from which we extracted data on the following variables: CO_2_ emissions, CHE per capita, DGGHE/CHE, DPHE/CHE, EHE/CHE, DGGHE/GGE, GDP per capita, and urbanization. For fragility, we obtained data from the World Bank classification, which listed the fragile countries from 2006 to 2025 [30]. For years prior to 2006, we assumed that countries retained their 2006 status. For immigration, we gathered data from the Department of Economic and Social Affairs of the United Nations, 2000–2020. The International Migrant Stock 2020 provides estimates of international migrants derived from the official data on the foreign-born or foreign population from 232 countries [26]. As the statistics were collected once every five years between 2000 and 2020, the data for the intervening years were linearly imputed within each five-year period. We constructed a panel dataset covering 21 years. Such an imputation may result in potential smoothing bias.

### Data analysis

Based on the income categories defined by the World Bank [31], we classified MENA countries into three groups, high-, middle- and low-income economies, as shown in Appendix A. We analyzed the trends in each mortality rate from 2000 to 2020 for each category of countries, as well as for the overall region. Annual growth rates of MMR, IMR, and U5MR were calculated for each country/territory between 2000 and 2020 using a log-linear form of the dependent variables against years.

To examine the relationship between mortality rates and potential determinants, we employed log–log regression models for the panel data from 2000 to 2020 [32]. Given that the sum of the three health financing indicators (e.g., DGGHE/CHE, DPHE/CHE, and EHE/CHE) always equals 100, we omitted DPHE/CHE from the regression model and used it as the reference group to avoid collinearity. Additionally, some countries did not receive any EHE with reported values of 0 for the EHE/CHE variable. We used the started logarithm form of EHE/CHE (log (EHE/CHE + 1)) as an independent variable in the model to avoid missing values while preserving the shape of the data. Such practice is consistent with previous studies [33, 34]. The final regression model for the panel data was specified as follows:

$$\begin{aligned} \log \left( {mortality_{it} } \right) = & \beta_{0} + \beta_{1} *\log \left( {\text{GDP per capita}} \right) + \beta_{2} *\log \left( {\text{CHE per capita}} \right) + \beta_{3} \\ & *\log \left( {\text{DGGHE/CHE}} \right) + \beta_{4} *\log \left( {\text{EHE/CHE + 1}} \right) + \beta_{5} *\log \left( {\text{DGGHE/GGE}} \right) + \beta_{6} \\ & *\log \left( {\text{Urbanization}} \right) + \beta_{7} *\log \left( {\text{Immigration}} \right) + \beta_{8} *\log \left( {CO_{2}\text{ emission}} \right) + \beta_{9} \\ & *Fragility + \alpha_{i} + \varepsilon_{it} \\ \end{aligned}$$

where ${mortality}_{it}$ represents MMR, IMR, and U5MR in *i*th country in time *t*, ${\alpha }_{i}$ is the country-specific effect, and ${\varepsilon }_{it}$ is the random error. We performed both random- and fixed-effects models and conducted Hausman tests to examine the difference between them. The results of the two models and the Hausman tests are presented in Appendix B. We reported the results from the fixed-effects model because the Hausman test was statistically significant (*p* < 0.05). Given the relatively small sample size of 21 countries, we set α = 0.1 as the threshold for statistical significance when reporting the results. All statistical analyses were conducted using R and Stata version 18.0 (StataCorp LP, Texas).

## Results

### Trend in MMR, IMR, and U5MR and growth rate

**Fig. 1:**
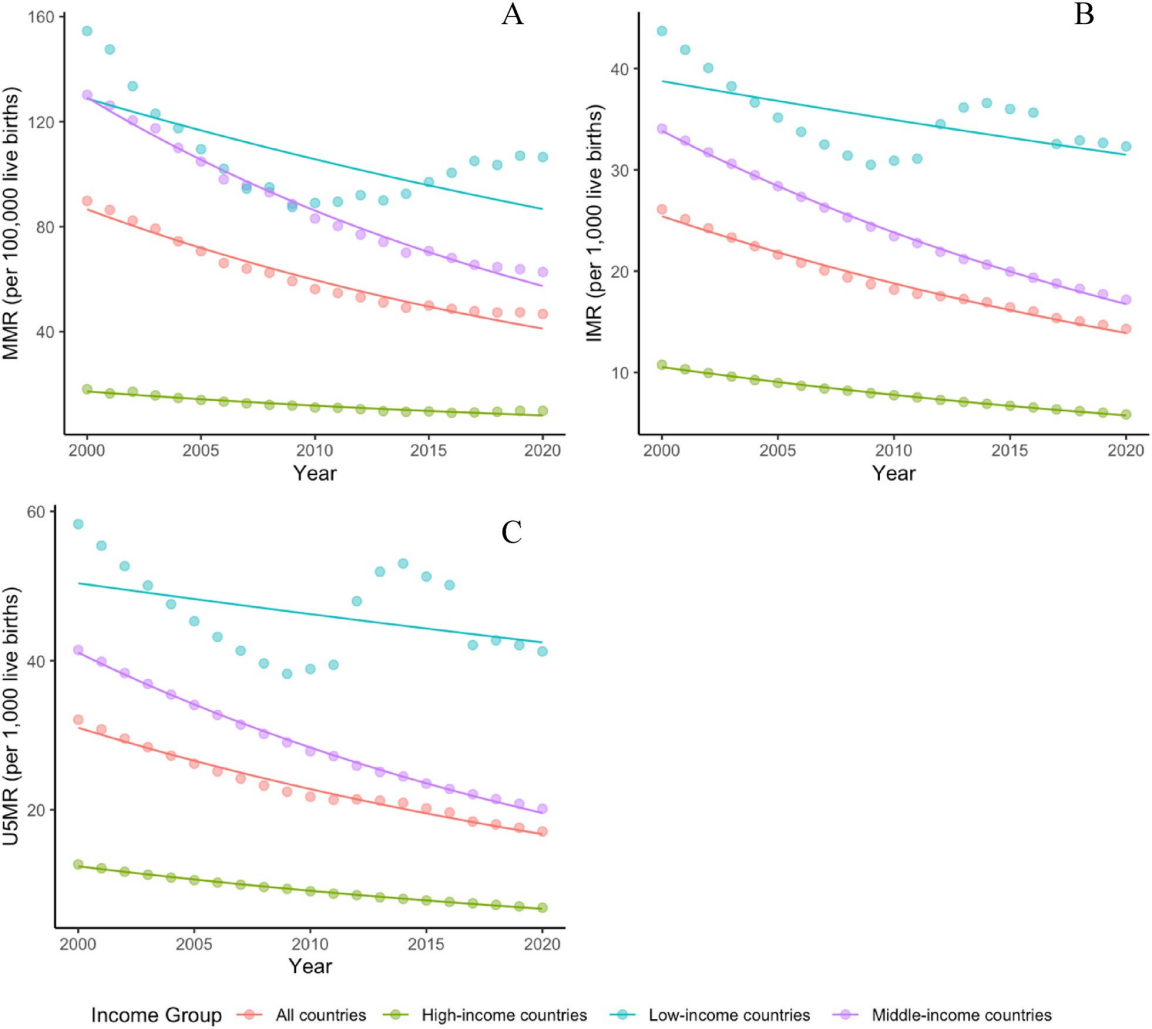
Average MMR, IMR, and U5MR in MENA countries from 2000 to 2020

The descriptive statistics for MCH mortality rates and potential determinants in 2000 and 2020, as well as their differences, are presented in Appendix C. Figure 1 illustrates the trend in MMR, IMR, and U5MR for the three income groups within the MENA region. In Fig. 1A, the average MMR across all MENA countries exhibited a decreasing trend, declining from 89.81 to 46.76 deaths per 100,000 live births over 21 years. As expected, there was substantial variation across the region when analyzed by country income group categories. High-income countries in the region had an average MMR steadily descending from 18.13 in 2000 to 9.88 deaths per 100,000 live births in 2020, with an annual reduction rate of 3.02% (*p* < 0.01). The middle-income countries experienced a statistically significant drop in MMR from 130.18 to 62.73 deaths per 100,000 live births over the same period, with an annual reduction rate of 3.73% (*p* < 0.01). The low-income countries also witnessed an overall downward trend in MMR. However, after a substantial decline between 2000 and 2009, MMR increased between 2010 and 2020. The annual reduction rate of MMR in low-income countries was 0.33% (*p* < 0.01).

Figure 1B displays the average IMR across all income groups, highlighting a consistent downward trend from 2000 to 2020. However, the IMR in low-income countries fluctuated during this period. Overall, there was a decrease of 11.80 deaths per 1,000 live births in the IMR within the region, declining from 26.10 in 2000 to 14.30 deaths per 1,000 live births in 2020. The IMR in high-income countries dropped from 10.75 to 5.85 deaths per 1,000 live births, with an annual reduction rate of 2.97% (*p* < 0.01). Middle-income countries in the MENA region experienced a more significant reduction in deaths, with an annual decrease rate of 3.46% (*p* < 0.01). However, in low-income countries, the IMR decreased from 43.70 per 1,000 live births in 2000 to 30.50 in 2009, then rose to 36.6 per 1,000 live births in 2014 before slightly declining to 32.3 per 1,000 live births by 2020. The overall annual reduction rate was 0.92% (*p* < 0.01).

The U5MR (Fig. 1C) followed a similar trend to that of IMR. The mean U5MR in the MENA area declined from 32.09 in 2000 to 17.09 deaths per 1,000 live births in 2020. The number in high-income countries slightly went down by 5.29 deaths per 1,000 live births during the 21 years. Middle-income countries had a higher reduction of 21.31 deaths per 1,000 live births over the period. The annual reduction rate of U5MR was 3.02% (*p* < 0.01) in high-income countries, compared to 3.64% (*p* < 0.01) in middle-income countries and 0.78% (*p* < 0.01) in low-income countries. Like IMR, U5MR in low-income countries experienced fluctuations throughout the period.

Table 2 shows that the annual growth rates of MMR, IMR, and U5MR varied considerably across countries. For MMR, Egypt had the highest reduction rate at 8.30%, while Libya experienced a slight annual increase of 0.019%. The annual growth rate of IMR across the MENA region ranged from − 5.84 to 1.19%, with only the Syrian Arab Republic exhibiting a positive rate. The annual change in U5MR ranged from − 5.85 to 2.23%, with Syria again displaying an upward trend. All other countries saw a reduction rate exceeding 1.50% annually.

**Table 2:** Annual growth rates of MMR, IMR, and U5MR from 2000 to 2020

	MMR (per 100,000 live births)			IMR (per 1,000 live births)			U5MR (per 1,000 live births)		
Country	2000	2020	Annual growth rate	2000	2020	Annual growth rate	2000	2020	Annual growth rate
Egypt	79	17	− 8.30%***	37.2	16.8	− 3.94%***	46.7	19.6	− 4.28%***
Qatar	28	8	− 7.96%***	10.7	4.8	− 3.61%***	12.5	5.7	− 3.55%***
West Bank and Gaza	62	20	− 6.68%***	25.3	13.2	− 3.25%***	30.4	15.3	− 3.40%***
Morocco	244	72	− 6.66%***	44.0	16.1	− 5.27%***	52.4	18.7	− 5.36%***
Malta	10	3	− 6.01%***	6.7	5.2	− 0.95%***	7.6	6.0	− 0.90%***
Iran	44	22	− 4.91%***	29.8	11.2	− 5.00%***	36.4	13	− 5.23%***
Israel	9	3	− 4.27%***	5.6	2.8	− 3.56%***	6.9	3.4	− 3.48%***
Algeria	159	78	− 3.95%***	35.6	19.6	− 2.95%***	41.7	22.9	− 2.95%***
United Arab Emirates	22	9	− 3.95%***	8.4	5.6	− 1.85%***	10.7	6.6	− 2.46%***
Djibouti	512	234	− 3.90%***	79.0	46.8	− 2.64%***	99.7	55.4	− 2.97%***
Tunisia	62	37	− 2.90%***	24.5	14.3	− 2.48%***	29.4	16.6	− 2.61%***
Bahrain	24	16	− 2.78%***	10.7	6.0	− 3.14%***	12.5	7.0	− 3.14%***
Kuwait	10	7	− 2.75%***	11.0	7.5	− 2.17%***	12.7	8.8	− 2.13%***
Iraq	117	76	− 2.50%***	35.6	21.4	− 2.58%***	44.4	25.4	− 2.85%***
Jordan	64	41	− 2.43%***	22.6	12.9	− 2.76%***	26.9	15.0	− 2.90%***
Yemen	275	183	− 1.91%***	67.8	45.8	− 1.75%***	93.5	59.7	− 1.95%***
Lebanon	32	21	− 1.89%***	17.0	7.2	− 4.47%***	19.8	8.5	− 4.44%***
Oman	20	17	− 1.82%***	14.1	8.9	− 1.88%***	16.4	10.4	− 1.85%***
Saudi Arabia	22	16	− 1.32%***	18.8	6.0	− 5.84%***	22.1	7.0	− 5.85%***
Syrian	34	30	1.05%	19.6	18.8	1.19%	23.1	22.8	2.23%*
Libya	57	72	1.89%***	24.1	9.5	− 5.00%***	28.1	11.1	− 4.98%***

The annual gross rate was derived from the given data points using a log-linear form. *p < 0.05; **p < 0.01, and ***p < 0.001

### Association between potential determinants and mortalities

Table 3 presents both unadjusted and adjusted coefficients for potential variables associated with mortality rates. In the unadjusted model, MMR was associated with all the selected independent variables. The relationships were statistically significant (*p* < 0.05).

**Table 3:** Association between MMR, IMR, U5MR, and selected indicators

Variables	Log(MMR)		Log(IMR)		Log(U5MR)	
	Unadjusted	Adjusted	Unadjusted	Adjusted	Unadjusted	Adjusted
Log(GDP per capita)	− 0.756***	− 0.121**	− 0.495***	− 0.076**	− 0.508***	− 0.138***
Log(CO_2_ emissions)	− 0.729***	0.265***	− 0.471***	0.001	− 0.485***	− 0.021
Log(CHE per capita)	− 0.879***	− 0.319***	− 0.567***	− 0.275***	− 0.575***	− 0.225***
Log(DGGHE/GGE)	− 1.070***	− 0.033	− 0.721***	− 0.052	− 0.746***	− 0.098**
Log(DGGHE/CHE)	− 1.699***	− 0.159*	− 1.161***	− 0.014	− 1.192***	0.037
Log(EHE/CHE + 1)	0.700***	0.007	0.437***	− 0.003	0.449***	− 0.002
Log(Urbanization)	− 2.445***	− 1.154***	− 1.703***	− 1.448***	− 1.783***	− 1.560***
Log(Immigration)	− 0.373***	− 0.276***	− 0.229***	− 0.018	− 0.231***	− 0.017
Fragility	1.109***	0.131**	0.660***	0.089***	0.700***	0.094***

*p < 0.10, **p < 0.05, ***p < 0.01. The coefficient for log (DGGHE/CHE) is the only one that is statistically significant at the 0.10 level but not at the 0.05 level. Please see Appendix D, Table S4

In the adjusted model, all variables, except DGGHE/GGE, DGGHE/CHE, and EHE/CHE, remained statistically significant at the 0.05 level. The estimated elasticity of MMR with respect to GDP per capita, CHE per capita, urbanization, and immigration was  − 0.121, − 0.319, − 1.154, and − 0.276, respectively. In comparison with DPHE/CHE, DGGHE/CHE elasticity of MMR was − 0.159. It was statistically significant at the 0.10 level but not at the 0.05 level. A 1% increase in DGGHE/CHE, compared to DPHE/CHE, was associated with a 0.159% reduction in MMR, suggesting a higher efficiency of public funding than private funding in reducing MMR. Additionally, the CO_2_ emission elasticity of MMR was 0.265, meaning that a 1% increase in CO_2_ emission was associated with a 0.265% increase in MMR (*p* < 0.01). A fragile status was associated with a 13.1% increase in MMR (*p* < 0.05).

In the adjusted model for IMR, four potential determinants were found to be statistically significant (*p* < 0.05). The elasticity of IMR in response to GDP per capita, CHE per capita, and urbanization was − 0.076, − 0.275, and − 1.448, respectively, all of which were statistically significant (*p* < 0.05). Every 1% increase in GDP per capita, CHE per capita, and urbanization was associated with a 0.076, 0.275, and 1.448% decrease in IMR, respectively. For fragility, the coefficient was estimated at 0.089, implying that fragility was correlated with a rise in IMR by 8.9%.

Comparable trends were also noted for U5MR, with five variables being statistically significant in the adjusted model (*p* < 0.05). In the adjusted model, the elasticity of U5MR with respect to GDP per capita, CHE per capita, DGGHE/GGE, and urbanization was − 0.138, − 0.225, − 0.098, and − 1.560, respectively. Additionally, the fragility status was associated with a 9.4% increase in U5MR (Table 3).

Due to the imputation of the variable of immigration, we conducted a sensitivity analysis using five panels of data from 2000, 2005, 2010, 2015, and 2020, where no imputation was made, and found similar results (Appendix D, Table S5) in terms of signs of the coefficients.

## Discussion

MMR, IMR, and U5MR declined in the MENA region, with varying rates across countries at different levels of economic development between 2000 and 2020. The highest reduction rates in mortality were observed in middle-income countries, followed by high-income countries and then low-income countries. In low-income and conflict-afflicted countries, the average rates initially decreased but fluctuated from 2009 onwards. Health financing, particularly total health expenditure per capita, is an important factor associated with mortality rates. Higher health expenditure per capita, higher socioeconomic status, progressive urbanization, and conflict-free status in a country were associated with lower mortality rates under the investigation. A greater share of government spending and a larger share of immigrants were associated with a lower MMR, but not with IMR and U5MR. Moreover, a stronger government commitment to health was associated with a lower under-5 mortality rate. Additionally, higher CO_2_ emissions were associated with a higher maternal mortality rate.

The study showed that there has been an overall decline in MCH-related mortality in the region, consistent with the global trend [35]. As previously reported, the reduction in MCH mortality can be attributable to multiple factors, including improvements in health financing, socioeconomic status, rising population awareness, and better women’s education [25, 36]. It is reasonable that the mortality reduction rate was the lowest in the high-income countries because their mortality rates were already very low, leaving little room for further improvement. For these countries, to further reduce MCH mortality, a more targeted approach toward vulnerable populations is needed. Countries with high mortality rates tend to have higher reduction rates for MCH mortality as they have much more potential for improvement [37]. However, this was not the case in MENA, where the greatest improvement was observed in the middle-income group instead of the low-income group. Such controversy may be explained by higher political instability in a few low-income MENA countries (e.g., Yemen and Syria), which impedes the progression of MCH outcome improvement.

Health expenditure plays a vital role in lowering mortality rates, consistent with other studies [38–40]. Our study shows that the health expenditure elasticities of MMR, IMR, and U5MR ranged from -0.319 to -0.225. The elasticities are similar to the estimates by Farag et al. [41]. A country with a higher total health spending often achieves better quality of care and higher coverage of essential services for pregnant women and children [42]. Specifically, for pregnant women and children, the adequacy of health funding is necessary for providing various MCH services, including prenatal care, basic essential obstetric care, postnatal care, and immunization [43]. It is estimated that a one percentage point increase in total health expenditure as a share of GDP was associated with an increase in the coverage of antenatal care, immunization, and skilled birth attendance by 1.49, 1.84, and 1.10 percentage points, respectively [44]. The overall investment in health would significantly advance the agenda to move countries towards UHC and improve population health outcomes. In countries where resource collection systems function effectively, governments should build on these systems to sustain health financing. In contrast, countries facing challenges in mobilizing health resources may need to adopt new and innovative strategies tailored to their specific contexts. Globally, instruments such as sin taxes and social bonds have gained increasing attention [45, 46].

Our findings show that government funding is more efficient in reducing MMR, compared with private funding, but not in reducing IMR and U5MR. Public spending is provided by the government or social health insurance schemes, while private spending comes from out-of-pocket spending, private health insurance, or private organizations. The source of funding, to some extent, determines how resources are allocated, which in turn affects efficiency [47]. The higher efficiency of public funding over private funding may lie in: (1) public funding focuses more on primary care and thus is more relevant to MCH. A study in the African region showed that the public sector, compared to the private funding sources, places a greater emphasis on preventive care for a wide range of populations, which directly affects MCH outcomes [48]; and (2) the public sector may leverage greater market power to enhance the efficiency of care delivery [13, 14, 48, 49]. For example, through centralized procurement of drugs and medical devices, the public sector can secure lower prices for medicines so as to increase medicine accessibility [49]. Given the importance of government funding in reducing maternal mortality and enhancing health system efficiency, MENA countries should continuously raise government financial support for evidence-based MCH services to improve associated outcomes. However, we did not find similar results for IMR and U5MR. Further exploration is needed to understand why IMR and U5MR are less sensitive to the government funding.

Our analysis revealed that mortality rates were negatively associated with GDP per capita after controlling for CHE. This is consistent with other studies [13, 32, 50, 51]. As an economic indicator, GDP per capita proves to be an exceptionally strong social factor influencing health [52]. The health status of the countries with higher GDP per capita benefits from the advancements in non-health sectors, such as education, transportation, agriculture, and water, sanitation, and hygiene (WASH) [53–55]. Taking women’s literacy as an example, it was reported that GDP per capita is closely associated with pregnant women’s health literacy, and women in high-income countries tend to be more knowledgeable about taking care of themselves and their children [56]. Every additional year of maternal education is associated with a 3.0% reduction in the U5MR [57]. The improvement of non-health sectors plays a crucial role in improving MCH outcomes.

We found that CO_2_, a measure of air pollution, was positively related to MMR, but not to child mortalities. Several studies have established that air pollution may exert direct health harm to pregnant women [58, 59], not only causing respiratory disease but also posing high risks for non-communicable diseases and mental disorders, such as hypertensive disorders, gestational diabetes, and depression. Specifically for pregnant women, air pollution was associated with a higher risk of maternal hypertensive disorders and placental abruption, leading to higher MMR [60]. However, we did not find that CO_2_ was associated with child mortalities, which is different from the findings reported previously [58, 59]. The smaller sample size of this study and the relatively short duration of children’s exposure to air pollution may be the reason for the difference.

Furthermore, we identified that some demographic factors were associated with MCH mortality, such as urbanization and immigration. Unlike other studies in Africa [13, 14], our analysis demonstrates that as countries become more urbanized, their MCH rates tend to be lower. Between 2000 and 2020, the urbanization rate in the MENA region increased from 58% to 65% [61]. While no unanimous findings have been reached regarding the relationship between urbanization and health outcomes, the impact of urbanization on health can be complex [62, 63]. In this study, we speculate that the increase in urbanization rate has significantly improved physical accessibility to health care for pregnant women and children, similar to the experience in China between 2004 and 2019 during a period of rapid urbanization [62]. Thus, they can receive timely treatment when falling ill.

For immigration, measured by the international migrant stock as a percentage of the total population, we found that it was associated with a reduction of MMR. Immigration serves as a proxy indicator of a country’s attractiveness. Countries that attract larger foreign-born populations are often wealthier, politically more stable, and equipped with stronger health systems [64]. These factors contribute to better health infrastructure, including health workforce attraction for pregnant women and broader access to prenatal and delivery services, which in turn can lead to a lower maternal mortality ratio. For instance, foreign doctors account for 73% of the total health workforce in Saudi Arabia, providing essential MCH services that reduce related mortalities [65]. Given the relatively high coverage of immunization among children in many high-income countries in the MENA region [66], the attractiveness may exert a higher impact on MMR than IMR and U5MR.

Mortality rates in high-income countries remained consistently low throughout the period, leaving limited room for further reduction. Achieving additional health improvements in these countries requires more resources at the national level [67]. It is critical for high-income countries to develop customized interventions aimed at continuously bringing the mortality rates down. For instance, conditional cash transfer (CCT) is an efficient strategy to improve coverage of MCH services. With support from the United Nations Children’s Fund (UNICEF), China implemented a CCT program targeting the use of maternal health services among pregnant women in western rural areas. Compared to non-participants, CCT participants had higher use of antenatal care, institutional delivery, and postnatal care [68]. Similarly, high-income countries can also implement such programs to incentivize pregnant women of vulnerable groups to seek healthcare services.

Since 2009, a few low-income countries have experienced an increase in mortality rates, at least until 2015, primarily due to conflicts in the MENA region. Yemen and Syria have been entangled in a series of wars and violence [69]. The crude death rate per 1000 people in Yemen rose from 5.65 to 6.82 from 2009 to 2022 [70], with an even worse situation in Syria [71]. The conflicts severely compromised the functions of the healthcare system, which failed to provide women and children with timely medical care. By 2013, 57% of public hospitals had sustained damage in Syria, with 36% rendered non-operational [72]. Meanwhile, the capacity of ambulances became inadequate due to attacks and frequent approximations by rebels [73, 74]. Furthermore, the shortage of doctors aggravated the inaccessibility of healthcare [73, 75]. By 2013, at least 160 doctors in Syria had been killed by anti-government forces, and hundreds were imprisoned for refusing to withhold healthcare services [76]. These threats compelled many doctors to flee the country. In Homs, a city in Syria, for instance, more than 50% of doctors had left [73]. Ongoing conflicts have weakened governance, contributing to societal instability [77]. The influx of refugees, driven by regional conflicts and instability, places a significant burden on the healthcare systems of host countries. This strain adversely affects health outcomes for both local populations and the displaced individuals [78]. Besides alleviating social unrest, Yemen and Syria should take actions to lower the mortality ratios, such as providing women with equal education opportunities as men through government programs to improve female education, and launching or expanding nutritional programs to improve the nutritional status of children. Additionally, such countries should also leverage external funding to enhance the domestic resources to raise the health awareness and deliver essential health services through, for example, conditional grants tied to the government’s financial commitment [79].

A few limitations of this study should be acknowledged. First, the available data on mortality rates and socioeconomic indicators in the MENA region were limited. When conducting the research, we were able to obtain data from 2000 to 2020, but not all countries provided consecutive data throughout the entire period. However, it had a minimal impact on our research as the missing values were limited. Second, we only had a limited number of potential determinants of mortality rates, and thus, the regression model may run into omitted variable issues. However, with the panel data, this concern was mitigated. Third, the interpretation of the findings should be approached with caution, as we cannot assure causality between mortalities and potential drivers [32]. Thus, we described their associations instead, which remains accurate in the analysis.

## Conclusions

MMR, IMR, and U5MR exhibited varying trends among the three income groups in the MENA region. Although the region as a whole experienced an overall reduction in all three mortality rates, the low-income countries followed the most challenging trajectory of mortality, which requires particular attention. Within the health system, health spending per capita is the major single determinant across the three maternal and child mortalities. Therefore, it is crucial for governments to develop and refine sound health financing mechanisms to raise resources for health to improve MCH outcomes, regardless of the sources of funding. Additionally, efforts should go beyond the health system to improve MCH outcomes in the low-income countries in the MENA region, addressing bottlenecks to boost their economic growth, improving political stability, and reducing armed conflicts is of particular importance.

## Supplementary Information

Additional file1 (DOCX 33 KB)

## Data Availability

All data used in this study are publicly available.

## Abbreviations

CCT: Conditional cash transfer
CHE: Current health expenditure
DGGHE/CHE: Domestic general government health expenditure as a share of current health expenditure
DGGHE/GGE: Domestic general government health expenditure as a share of general government expenditure
DPHE/CHE: Domestic private health expenditure as a share of current health expenditure
EHE/CHE: External health expenditure as a share of current health expenditure
GDP: Gross domestic product
IMR: Infant mortality rate
MCH: Maternal and child health
MENA: Middle East and North Africa
MMR: Maternal mortality rate
U5MR: Under-5 mortality rate
UHC: Universal health coverage
UNICEF: United Nations Children’s Fund
WASH: Water, sanitation, and hygiene
WDI: World development indicators
