# Supplementary material for: The maternal and child mortality in the Middle East and North Africa between 2000 and 2020: the role of health financing
Source: Glob Health Res Policy. 2025 Oct 30;10:57. doi: 10.1186/s41256-025-00459-1 (PMC12573875; doi:10.1186/s41256-025-00459-1)
Supplement: Supplementary file 1 — Additional file1 (DOCX 33 KB) [file 41256_2025_459_MOESM1_ESM.docx]

**Appendix**

**Appendix A. Classification of countries in the MENA region**

Table S1 Classification of countries in the MENA region

| Group | Number of countries | Countries |
| --- | --- | --- |
| High-income countries | 8 | Bahrain, Israel, Kuwait, Oman, Qatar, Saudi Arabia, Malta and the United Arab Emirates |
| Middle-income countries | 11 | Algeria, Egypt, Iran, Iraq, Jordan, Lebanon, Libya, Morocco, the West Bank and Gaza, Djibouti, and Tunisia |
| Low-income countries | 2 | Syrian, Yemen |

**Appendix B: Random- and fixed-effects models examining the association between MCH mortality rates and potential determinants**

Table S2 Potential determinants of mortality rates using random- and fixed-effects models

| Variables | Log(MMR) | | Log(IMR) | | Log(U5MR) | | |  |
| --- | --- | --- | --- | --- | --- | --- | --- | --- |
|  | Random-effects model | Fixed-effects model | Random-effects model | Fixed-effects model | | Random-effects model | Fixed-effects model | |
| Log(GDP per capita) | -0.125** | -0.121** | -0.082** | -0.076** | | -0.142*** | -0.138*** | |
| Log(CO2 emissions) | 0.215*** | 0.265*** | -0.024 | 0.001 | | -0.036 | -0.021 | |
| Log(CHE per capita) | -0.354*** | -0.319*** | -0.317*** | -0.275*** | | -0.274*** | -0.225*** | |
| Log(DGGHE/GGE) | -0.043 | -0.033 | -0.070 | -0.052 | | -0.117*** | -0.098** | |
| Log(DGGHE/CHE) | -0.115 | -0.159* | 0.045 | -0.014 | | 0.100* | 0.037 | |
| Log(EHE/CHE+1) | 0.018 | 0.007 | -0.006 | -0.003 | | -0.007 | -0.002 | |
| Log(Urbanization) | -0.737** | -1.154*** | -0.772*** | -1.448*** | | -0.845*** | -1.560*** | |
| Log(Immigration) | -0.229*** | -0.276*** | 0.015 | -0.018 | | 0.029 | -0.017 | |
| Fragility | 0.138*** | 0.131** | 0.094*** | 0.089*** | | 0.096*** | 0.094*** | |
| Hausman test | 26.05 (p=0.0020) | | 35.54 (p=0.0000) | | 32.95 (p=0.0001) | | |  |

Note: * p<0.10, **p<0.05, ***p<0.01

**Appendix C. Descriptive analysis of independent variables**

Table S2 shows the changes in the selected independent variables between 2000 and 2020. Among the 9 indicators mentioned above, significant changes were observed for two of them, CHE per capita and DGGHE/GGE. The mean CHE per capita of all the MENA countries significantly increased from US$326.83 in 2000 to US$991.51 in 2020. And the mean of DGGHE/GGE significantly rose by approximately 3% over the twenty years. However, the differences in the means of the remaining indicators were statistically insignificant.

Table S3 Descriptive statistic

| Variable | Mean$\pm$SD (2000) | Mean$\pm$SD (2020) | Difference |
| --- | --- | --- | --- |
| GDP per capita | 8853.46$\pm$9803.65 | 14336.48$\pm$16213.91 | 5483.02 |
| CO_2_ emissions | 10.28$\pm$11.86 | 9.23$\pm$9.26 | -1.05 |
| CHE per capita | 326.83$\pm$362.02 | 991.51$\pm$1004.94 | 664.68* |
| DGGHE/CHE | 54.21$\pm$17.45 | 60.79$\pm$18.72 | 6.58 |
| DGGHE/GGE | 7.78$\pm$2.54 | 10.72$\pm$4.21 | 2.94* |
| DPHE/CHE | 45.26$\pm$17.38 | 37.90$\pm$17.58 | -7.36 |
| EHE/CHE | 0.68$\pm$1.68 | 1.32$\pm$2.78 | 0.64 |
| Urbanization | 70.97$\pm$19.27 | 77.21$\pm$17.94 | 6.24 |
| Immigration | 21.81$\pm$24.05 | 26.91$\pm$29.86 | 5.10 |

Note: *p<0.05

**Appendix D: Sensitivity analysis of the regression models**

Table S4 Association between MMR, IMR, U5MR and selected indicators using 5% as the significance level

| Variables | Log(MMR) | | Log(IMR) | | Log(U5MR) | |
| --- | --- | --- | --- | --- | --- | --- |
|  | Unadjusted | Adjusted | Unadjusted | Adjusted | Unadjusted | Adjusted |
| Log(GDP per capita) | -0.756*** | -0.121* | -0.495*** | -0.076* | -0.508*** | -0.138*** |
| Log(CO2 emissions) | -0.729*** | 0.265*** | -0.471*** | 0.001 | -0.485*** | -0.021 |
| Log(CHE per capita) | -0.879*** | -0.319*** | -0.567*** | -0.275*** | -0.575*** | -0.225*** |
| Log(DGGHE/GGE) | -1.070*** | -0.033 | -0.721*** | -0.052 | -0.746*** | -0.098* |
| Log(DGGHE/CHE) | -1.699*** | -0.159 | -1.161*** | -0.014 | -1.192*** | 0.037 |
| Log(EHE/CHE+1) | 0.700*** | 0.007 | 0.437*** | -0.003 | 0.449*** | -0.002 |
| Log(Urbanization) | -2.445*** | -1.154*** | -1.703*** | -1.448*** | -1.783*** | -1.560*** |
| Log(Immigration) | -0.373*** | -0.276*** | -0.229*** | -0.018 | -0.231*** | -0.017 |
| Fragility | 1.109*** | 0.131* | 0.660*** | 0.089** | 0.700*** | 0.094** |

Note: * p<0.05, **p<0.01, ***p<0.001

Table S5 Association between MMR, IMR, U5MR and selected indicators using 5-year panel

| Variables | Log(MMR) | | Log(IMR) | | Log(U5MR) | |
| --- | --- | --- | --- | --- | --- | --- |
|  | Unadjusted | Adjusted | Unadjusted | Adjusted | Unadjusted | Adjusted |
| Log(GDP per capita) | -0.764*** | -0.198 | -0.502*** | 0.009 | -0.515*** | -0.008 |
| Log(CO2 emissions) | -0.711*** | 0.306* | -0.463*** | 0.025 | -0.475*** | 0.014 |
| Log(CHE per capita) | -0.874*** | -0.352** | -0.570*** | -0.417*** | -0.578*** | -0.415*** |
| Log(DGGHE/GGE) | -1.136*** | 0.045 | -0.744*** | 0.132 | -0.764*** | 0.138 |
| Log(DGGHE/CHE) | -1.609*** | -0.348 | -1.082*** | -0.236 | -1.110*** | -0.226 |
| Log(EHE/CHE+1) | 0.670*** | -0.061 | 0.416*** | -0.048 | 0.428*** | -0.05 |
| Log(Urbanization) | -2.442*** | -0.608 | -1.714*** | -1.325*** | -1.789*** | -1.412*** |
| Log(Immigration) | -0.369*** | -0.168 | -0.230*** | 0.019 | -0.232*** | 0.022 |
| Fragility | 1.075*** | -0.019 | 0.636*** | 0.013 | 0.672*** | 0.013 |

Note: * p<0.10, **p<0.05, ***p<0.01
